# Kostenanalyse stationärer und ambulanter intravenöser Antibiotikatherapie periprothetischer Gelenkinfektionen: Eine Simulation

**DOI:** 10.1007/s00132-020-03889-6

**Published:** 2020-02-19

**Authors:** Christoph Kolja Boese, Philipp Lechler, Michael Frink, Michael Hackl, Peer Eysel, Christian Ries

**Affiliations:** 1 Klinik für Orthopädie und Unfallchirurgie, Universitätsklinik Köln (AöR), Joseph-Stelzmann-Str. 9, 50924 Köln, Deutschland; 2 Klinik für Unfall- und Handchirurgie, Kreiskliniken Altötting, Altötting, Deutschland; 3 Klinik für Orthopädie und Unfallchirurgie, Universitätsklinikum Gießen und Marburg, Marburg, Deutschland; 4 Klinik für Orthopädie, Universitätsklinikum Hamburg-Eppendorf, Martinistraße 52, 20246 Hamburg, Deutschland

**Keywords:** Gesundheitskosten, Gesamtkosten, Reinerlös, Opportunitätskosten, Kostenkalkulation, Healthcare costs, Total costs, Pure proceeds, Opportunity costs, Cost calculation

## Abstract

**Hintergrund:**

Die parenterale Antibiotikagabe im Rahmen der Therapie von periprothetischen Infektionen erfordert in der Regel eine stationäre Behandlung und geht mit hohen Kosten einher.

**Fragestellung:**

Es wurden tatsächliche stationäre Behandlungskosten („inpatient parenteral antibiotic therapy“ [IPAT]) mit simulierten Kosten einer ambulanten Behandlung („outpatient parenteral antibiotic therapy“ [OPAT]) von Patienten mit periprothetischen Gelenkinfektionen verglichen. Die Auswertung erfolgte aus Perspektive der Kostenträger (gesetzliche Krankenversicherung [GKV]) und Leistungserbringer (Krankenhäuser).

**Material und Methoden:**

Die Analyse und Simulation erfolgten auf Grundlage einer ICD-10 (Internationale statistische Klassifikation der Krankheiten und verwandter Gesundheitsprobleme, 10. Revision) für das Behandlungsjahr 2015 mit der Diagnose T84.

**Ergebnisse:**

Die simulierte Reduktion von 159 Bettentagen bei den in die Studie eingeschlossenen 12 Patienten erbrachte aus Sicht der Kostenträger eine Reduktion der Gesamtkosten um >18.000 €. Aus Perspektive der Leistungserbringer verbesserte sich der Reinerlös um >22.000 €. Die Gesamtkosten der OPAT für den Kostenträger beliefen sich auf >57.000 €. Für den Leistungserbringer zeigte sich in der Differenz von Poliklinikerlös und -kosten der OPAT ein Verlust von >1500 €.

**Diskussion:**

Die OPAT ist für Leistungserbringer insgesamt finanziell vorteilhaft. Weitere Vorteile durch Opportunitätskosten erscheinen interessant. Für den Kostenträger ist die OPAT insbesondere durch die ambulanten Medikamentenkosten mit einem finanziellen Mehraufwand verbunden. Der niedergelassene Sektor sollte durch die anzunehmende Mehrbelastung ebenso wie der anzunehmende Patientenkomfort bedacht werden.

## Abstract (en)

**Background:**

Parenteral antibiotic administration in the treatment of periprosthetic joint infections (PJI) often requires inpatient settings. This is associated with significant costs to the healthcare system.

**Objective:**

The costs of inpatient parenteral antibiotic treatment (IPAT) and simulated costs of outpatient parenteral antibiotic treatment (OPAT) were compared in patients with PJI. Evaluations were carried out from the perspectives of cost bearers (insurances) and healthcare providers (hospitals).

**Material and methods:**

The analysis and simulations were performed for all cases with the ICD-10 diagnosis T84 in the treatment year 2015.

**Results:**

The simulated reduction of 159 bed-days in the 12 patients included in the study resulted in a reduction of the total costs of>18,000 € from the perspective of the health insurance. From the perspective of the hospitals the pure proceeds were improved by >22,000 €. The total costs of OPAT were >57,000 € for the health insurance. For hospitals the difference of policlinic proceeds and costs of OPAT showed a loss of >1500 €.

**Conclusion:**

For hospitals the OPAT is overall financially advantageous. Further advantages due to opportunity costs seem to be interesting. For cost bearers OPAT is associated with an additional financial expenditure, particularly due to costs of outpatient medication. The private sector should be considered due to the assumed additional burden as well as the assumed patient comfort.

Durch die steigende Anzahl der Implantationen von Gelenkprothesen ist in Zukunft auch mit einem Anstieg von periprothetischen Infektionen zu rechnen. Die parenterale Antibiotikagabe bei periprothetischer Infektion erfordert in der Regel eine stationäre Behandlung und geht mit hohen Kosten einher. Der Ansatz einer ambulanten parenteralen Antibiotikagabe wie in den USA und Großbritannien ist in der Bundesrepublik Deutschland bisher nicht etabliert. Anhand einer Analyse von stationärer und Simulation ambulanter Antibiotikagabe sollen Rückschlüsse auf die Kosten gezogen und Empfehlungen ausgesprochen werden.

Die parenterale Antibiotikagabe bei periprothetischer Infektion („periprosthetic joint infections“ [PJI]) erfordert in der Regel eine stationäre Behandlung („inpatient parenteral antibiotic therapy“ [IPAT]). Die hieraus resultierenden Kosten für das Gesundheitssystem sind nicht unerheblich. Es können direkte Kosten (z. B. stationäre Behandlung) von indirekten Kosten (z. B. Arbeitsausfall der Patienten, Opportunitätskosten durch blockierte Kapazitäten) abgegrenzt werden [12]. Entgegen den Vorbildern aus den USA und Großbritannien gibt es in Deutschland bisher kein etabliertes System aus ambulanten Therapiezentren für die parenterale Gabe von Antibiotika („outpatient parenteral antibiotic therapy“ [OPAT]). Erste Apotheken bieten jedoch auch in Deutschland eine Heimtherapie mit einem Teil der relevanten Dienstleistungen und Verbrauchsmaterialien an. Die bisherigen Studien zum Kostenvergleich der OPAT und IPAT konnten außerhalb Deutschlands fast ausschließlich eine Kostenersparnis durch OPAT nachweisen [2, 9]. Mehrere Studien konnten neben der Wirksamkeit eine geringe Komplikationsrate belegen, die mit stationären Behandlungen vergleichbar waren [5, 7]. Des Weiteren zeigte sich eine hohe Patientenzufriedenheit [1, 13]. Somit erscheint die OPAT nicht nur aus medizinischer, sondern auch aus subjektiver Patientensicht vertretbar. Wenngleich die OPAT multipel einsetzbar ist, konnte auch explizit für Osteomyelitiden und PJI eine erfolgreiche Anwendung beobachtet werden [15]. Die Daten für PJI sind jedoch gering [4, 10, 16]. Bisher wurde keine Studie aus Deutschland zum Thema der möglichen Kostenreduktion durch OPAT anstelle von IPAT für die Behandlung von PJI der Hüft- und Kniegelenke durchgeführt.

In der vorliegenden Arbeit sollen daher die tatsächlichen Kosten der IPAT anhand einer Kohorte von Patienten mit PJI analysiert werden. In einem zweiten Schritt erfolgt eine Kostensimulation einer OPAT für dieses Patientenkollektiv. Abschließend sollen anhand der Kostenanalyse beider Optionen Rückschlüsse gezogen und Empfehlungen ausgesprochen werden. Dabei sollen die Kosten jeweils aus 2 Perspektiven 1. Kostenträgerperspektive (gesetzliche Krankenversicherungen [GKV]) und 2. Leistungserbringerperspektive (Krankenhäuser) – verglichen werden.

## Methodik

### Erhebung der klinischen Daten

Durch das klinikinterne Controlling wurde auf Basis der Abrechnungsdaten eine Liste aller stationären Behandlungsfälle mit der Diagnose T84 (Komplikationen durch orthopädische Endoprothesen, Implantate oder Transplantate) erstellt. Die Verschlüsselung der Diagnose erfolgte auf der ICD-10 GM (Internationale statistische Klassifikation der Krankheiten und verwandter Gesundheitsprobleme, 10. Revision, German Modification). Diese IDC-10-basierte Fallsuche erbrachte für das abgerechnete und komplett zur Verfügung stehende Jahr 2015 eine Gesamtzahl von 347 Fällen.

Ein Antrag für die Durchführung der retrospektiven Datenauswertung wurde der zuständigen Ethikkommission vorgelegt. Aufgrund des retrospektiven Charakters war kein Einverständnis der Patienten erforderlich.

### Patientenkollektiv zur Kalkulationsgrundlage

Die Diagnosen T84.5 (Infektion und entzündliche Reaktion durch eine Gelenkendoprothese), T84.6 (Infektion und entzündliche Reaktion durch eine interne Osteosynthesevorrichtung [jede Lokalisation]) sowie T84.7 (Infektion und entzündliche Reaktion durch sonstige orthopädische Endoprothesen, Implantate oder Transplantate) wurden im Verfahrensjahr 2015 insgesamt in 131 Fällen codiert. Für die Identifikation der kalkulierbaren Fälle wurden Patienten anhand definierter Kriterien ein- bzw. ausgeschlossen (Tab. 1). Zwei zusätzliche gültige Fälle konnten im Rahmen der Aktenrecherche zweier ausgeschlossener Fälle identifiziert werden. Diese wurden in der initialen Suche nicht erkannt, da die Aufnahmen noch im Jahr 2014 erfolgten und die Fälle dem Verfahrensjahr 2014 zugerechnet wurden.

**Table 1:** 

Ausgeschlossene Patienten	Anzahl (*n*)
Alter <18 Jahre	1
Aufenthaltsdauer <7 Tage	33
„Komplexe Fälle“	29
Infektion anderer Implantate als Hüft- oder Knie-TEP	26
Keine Antibiotikabehandlung	15
Kein Infekt	5
Orale Antibiotikabehandlung	4
Verlegung in eine andere Klinik	8
Summe	121

*TEP* Totalendoprothese

### Tatsächliche Kosten der „inpatient parenteral antibiotic therapy“

#### Kostenträger

Die Kosten des stationären Aufenthaltes aus Perspektive der Kostenträger entsprechen den Erlösen der Krankenhäuser. Für den stationären Aufenthalt werden sie mit den jeweiligen DRG (Diagnosis Related Groups) abgebildet. Die Tab. 2 zeigt die identifizierten Fälle und Tab. 3 die zugehörigen Grundlagen zur Erlöskalkulation nach DRG. Es werden die untere, mittlere und obere Grenzverweildauer (UGVD, MVD, OGVD) sowie die Abschläge und Zusatzentgelte dargestellt. Der erste Tag mit Abschlag ist die UGVD −1. Der erste Tag mit zusätzlichem Entgelt ist die OGVD +1. Für Aufenthalte zwischen UGVD und OGVD wird eine fixe Bewertungsrelation angegeben. Für die Unter- oder Überschreitung werden pro Tag Ab‑/Zuschläge mit einem Wert der Bewertungsrelation berechnet. Die Summe der Bewertungsrelationen ergibt pro Fall das Relativgewicht. Die individuellen Erlöse wurden als Multiplikation des Bundesbasisfallwertes (BBFW) und des Relativgewichts errechnet; der BBFW betrug 3231,20 €.

**Table 2:** 

Fall	Alter (Jahre)	DRG	Grund der Behandlung
01	73	I03B	Girdlestone-Situation nach HTEP-Infektion
02	76	I12A	Frühinfekt nach HTEP
03	65	I12A	Spätinfekt nach KTEP
04	75	I04Z	Spätinfekt nach HTEP
05	75	I03B	Chronischer Spätinfekt nach HTEP
06	75	I12C	Chronischer Infekt nach KTEP
07	63	I12B	Spätinfekt nach KTEP
08	76	I01Z	Chronischer Spätinfekt nach HTEP
09	73	I03B	Girdlestone-Situation und Osteomyelitis nach HTEP-Infektion
10	84	I12B	Frühinfekt nach HTEP
11	65	I12A	Frühinfekt nach HTEP
12	71	I01Z	Chronischer Spätinfekt nach HTEP

*DRG* Diagnosis Related Groups, *HTEP* Hüfttotalendoprothese , *ICD-10* Internationale statistische Klassifikation der Krankheiten und verwandter Gesundheitsprobleme, 10. Revision, *IPAT* „inpatient parenteral antibiotic therapy“, *KTEP* Knietotalendoprothese

**Table 3:** 

DRG	Fälle (*n*)	UGVD in Tagen	BR	MVD in Tagen	BR	OGVD in Tagen	BR	ZE OGVD (€)
I01Z	2	11	0,326	33,0	6,033	50	0,076	245,57
I03B	3	6	0,346	18,5	3,729	33	0,079	255,27
I04Z	1	5	0,318	16,2	3,544	28	0,075	242,34
I12A	3	7	0,354	21,6	3,484	39	0,080	258,50
I12B	2	5	0,314	15,5	2,302	30	0,071	229,42
I12C	1	3	0,293	9,2	1,391	19	0,067	216,49

*BR* Bewertungsrelation, *DRG* Diagnosis Related Groups; *MVD* Mindestverweildauer, *OGVD* obere Grenzverweildauer, *UGVD* untere Grenzverweildauer, ZE Zusatzentgelte

Zusatzentgelte bzw. Tagessätze über der OGVD wurden als Faktor der Bewertungsrelation und des Basisfallwertes des Jahres 2015 kalkuliert

#### Leistungserbringer

Diese Perspektive betrachtet die detaillierten Kosten anhand der einzelnen Leistungen, die der Krankenhausträger erbringt. Die Kalkulation der stationären Behandlungskosten beruht auf den durch das Institut für Qualitätssicherung und Transparenz im Gesundheitswesen (IQTiG) definierten Parametern und dem Handbuch des Instituts für das Entgeltsystem im Krankenhaus (InEK) zur Kalkulation von Behandlungskosten [6]. Es wurde anhand von Kostenstellen und Kostenarten eine individuelle Aufstellung der Kosten jedes Falles erstellt. Die Kosten für operative Maßnahmen und intensivmedizinische Aufenthalte etc. fielen grundsätzlich im Zeitraum vor einer möglichen Entlassfähigkeit an.

Es wurde ein Tagessatz für Gemeinkosten inklusive Personal (Pflege, Ärzte, Hausmeister, Sicherheitsdienst etc.), Verwaltung, Technik, Gebäude, Nebenkosten etc. berechnet. Personenbezogene Kosten durch z. B. Konsile, Laboruntersuchungen, radiologische Diagnostik, zentralvenöse Zugänge (PICC [„peripherally inserted central venous catheter“], ZVK [zentraler Venenkatheter] etc.), Medikamente (inklusive Antibiotika) wurden auf Fallbasis kalkuliert. Die Kalkulation erfolgte auf Grundlage der internen Daten, die durch das Controlling der Uniklinik bereitgestellt wurden.

### Grundlagen zur Kostensimulation der „outpatient parenteral antibiotic therapy“

Die Kostensimulation einer OPAT für die identifizierten Fälle erforderte zunächst die Erstellung eines Modells. In Anlehnung an die Community IntraVenous Antibiotic Study (CIVAS) von Minton et al. wird in dieser Arbeit die Selbstmedikation als Instrument der OPAT besprochen [8].

Kriterien für eine simulierte frühere Entlassfähigkeit wurden retrospektiv anhand der Aktenlage geprüft [3, 11, 14]. Als frühester möglicher Zeitpunkt der Entlassung wurden folgende Kriterien definiert:

- 5. Tag nach letzter Operation,
- trockene Wunde (wenn nicht anders dokumentiert),
- Tag nach letzter medizinischer Intervention/Diagnostik (außer Röntgen/Labor).

Insgesamt wurden 12 Fälle identifiziert (Tab. 2), welche die Einschlusskriterien erfüllten. Die mittlere Aufenthaltsdauer betrug 41 Tage (Spanne: 19 bis 84 Tage). Das mittlere Alter lag bei 72,6 Jahren (Spanne: 63 bis 84 Jahre).

Im Modell wurden medizinische Kontrollen in einer universitären Poliklinik durchgeführt. Die notwendigen Kosten für Labor und radiologische Diagnostik im Behandlungsverlauf wurden aus beiden Perspektiven als kostenneutral angenommen. Kosten der Einweisung in die OPAT und der pflegerischen Betreuung durch die beauftragte Apotheke werden in der Regel kostenfrei durch die Apotheken angeboten und fanden daher keinen Eingang in die Simulation. Eine Kalkulation von Opportunitätskosten (z. B. durch erneute Belegung frei werdender Betten) erfolgte nicht.

Die Kalkulation zur Berechnung der vermeidbaren Kosten der stationären Aufenthalte begann ab dem Tag der simulierten Entlassfähigkeit und basierte auf den eingesparten Bettentagen.

### Simulierte Kosten der „inpatient parenteral antibiotic therapy“

#### Kostenträger

Diese Perspektive der gesetzlichen Krankenversicherungen betrachtete die abgerechneten Kosten auf Basis der DRG. Dies entsprach den Erlösen des Krankenhauses.

#### Leistungserbringer

Die tatsächlich auftretenden Kosten für Krankenhäuser wurden durch Multiplikation der verkürzten Liegedauer (Bettentage) und der pauschalen fallspezifischen Tagessätze kalkuliert.

### Simulierte Kosten für „outpatient parenteral antibiotic therapy“

#### Kostenträger

Die Kosten der ambulanten ärztlichen Kontrollen wurden auf Basis von Pauschalen mit quartalsweiser Abrechnung für die Poliklinik kalkuliert. Die Pauschale für 1 Quartal betrug im Jahr 2015 92 €. Alternative Behandlungsmöglichkeiten und Transportkosten wurden nicht kalkuliert. Die Therapiedauer wurde entsprechend den eingesparten Bettentagen simuliert. Die Abrechnung erfolgte pauschaliert pro Quartal zuzüglich der rezeptierten Medikamente und Materialien. Die Medikamentenkosten wurden entsprechend der Hilfsmitteltaxe kalkuliert.

#### Leistungserbringer

Die Kalkulation der Kosten aus Sicht der Leistungserbringer einer Poliklinik basierte auf den Kostenstellen- und Kostenartenrechnungen vergleichbar mit dem stationären Aufenthalt. Die Daten wurden vom internen Controlling bereitgestellt. Die allgemeinen Kosten pro Vorstellung in der Poliklinik betrugen: Personal 46 €, Sachbedarf 3 € und Overhead 26 € (Summe 75 €).

Für die Kalkulation der simulierten OPAT-Behandlung wurden 2 verschiedene Kontrollregime in Abhängigkeit der Medikation verwendet. Die Häufigkeit der Vorstellungen in der Poliklinik wurde entsprechend den zu verabreichenden Antibiotika kalkuliert (Vancomycin: 3‑mal/Woche; andere Antibiotika: 1‑mal/Woche).

### Gesamtkosten der „outpatient parenteral antibiotic therapy“

Aus beiden Perspektiven wurden die Kosten der tatsächlichen IPAT den Kosten der simulierten OPAT gegenübergestellt. Die Gesamtkosten der OPAT bildeten sich aus den Kosten der Verkürzung der IPAT und der sich anschließenden simulierten OPAT.

Die Gesamtkosten der OPAT aus Perspektive der Kostenträger setzten sich aus einer Reduktion von stationären Behandlungskosten, zusätzlichen Kosten für ambulante Behandlungen und Kosten für Antibiotika zusammen. Nicht berücksichtigt wurden Transportkosten und Behandlungskosten für z. B. Physiotherapie.

## Ergebnisse

### Tatsächliche Kosten der „inpatient parenteral antibiotic therapy“

#### Kostenträger

Die Kosten aus Perspektive der gesetzlichen Krankenkassen entsprechen den Erlösen des Krankenhauses (Tab. 4, Spalte 4). Die Gesamtkosten beliefen sich auf 196.260,57 €.

**Table 4:** 

Fallnr	DRG	Kosten [K] (€)	Erlös [E] (€)	Diff. [K–E] (€)	VD in Tagen	OGVD in Tagen	Diff. [VD–OGVD] in Tagen
01	I03B	40.440,18	47.539,76	7099,58	66	47	19
02	I12A	23.541,59	17.594,41	−5947,18	66	39	27
03	I12A	20.246,01	11.116,78	−9129,23	35	39	−4
04	I04Z	13.932,11	11.308,23	−2623,88	26	28	−2
05	I03B	23.074,57	14.754,72	−8319,85	40	33	7
06	I12C	12.411,70	5721,10	−6690,60	25	19	6
07	I12B	12.662,25	7345,24	−5317,01	21	30	−9
08	I01Z	45.209,26	27.495,16	−17.714,10	84	50	34
09	I03B	10.335,46	11.898,53	1563,07	19	33	−14
10	I12B	16.010,48	7345,24	−8665,24	26	30	−4
11	I12A	10.920,91	11.116,78	195,87	22	39	−17
12	I01Z	23.498,87	23.024,62	−474,25	61	50	11
*Summe*	*–*	*252.283,39*	*196.260,57*	*−56.022,82*	*–*	*–*	*–*

Mit angegeben sind die tatsächliche Verweildauer sowie die jeweilige OGVD mit sich daraus ergebender Differenz. Die Begriffe Erlös/Kosten beziehen sich auf die Krankenhausperspektive.

*Diff.* Differenz, *DRG* Diagnosis Related Groups, *Fallnr* Fallnummer, *VD* Verweildauer, *OGVD* obere Grenzverweildauer

#### Leistungserbringer

Die durch auf Basis der DRG erwirtschafteten Beträge wurden den tatsächlichen Kosten gegenübergestellt. In 3 Fällen wurde ein Überschuss erwirtschaftet. In 9 Fällen resultierten Verluste. Die Differenz von Erlösen zu Kosten belief sich auf −56.022,82 €. In Bezug auf die Kosten von 252.283,39 € entspricht der Fehlbetrag einem Anteil von 22 % (Tab. 4, Spalten 3–5).

### Simulierte frühere Entlassung

Durch die OPAT wurde eine Reduktion von 159 Bettentagen simuliert (Tab. 5). Die Veränderung der Krankenhauserlöse bzw. Kosten für Versicherungen zeigen Veränderungen nur für Fälle oberhalb der OGVD. In keinem Fall wurde die UGVD unterschritten.

**Table 5:** 

Fallnr	Erlös (€)	Diff. [VD–OGVD] in Tagen	Tagessatz nach DRG (€)	VD ab Entlassfähigkeit in Tagen	Reduktion Erlös (€)
01	47.539,76	19	904,74	5	4523,70
02	17.594,41	27	258,50	15	3877,50
03	11.116,78	−4	258,50	6	–
04	11.308,23	−2	242,34	4	–
05	14.754,72	7	255,26	15	1786,82
06	5721,10	6	216,49	17	1298,94
07	7345,24	−9	229,42	15	–
08	27.495,16	34	245,57	16	3929,12
09	11.898,53	−14	255,26	13	–
10	7345,24	−4	229,42	14	–
11	11.116,78	−17	258,50	14	–
12	23.024,62	11	245,57	25	2701,27
*Summe*	*196.260,57*	*–*	*–*	*159*	*18.117,35*

*Diff.* Differenz, *DRG* Diagnosis Related Groups, *Fallnr* Fallnummer, *VD* Verweildauer

^a^Bei VD unter der OGVD kommt es zu keiner Erlösreduktion

### Simulierte Kosten der „inpatient parenteral antibiotic therapy“

Die frühere Entlassung reduzierte die Kosten der stationären Behandlung für Krankenkassen um 18.117,35 €. Dies ist einerseits eine Kostenreduktion für die Krankenversicherung und andererseits eine Erlösreduktion für die Krankenhäuser. Für Krankenhäuser stand der Erlösreduktion eine Kostenreduktion von 40.713,60 € gegenüber. Der Fehlbetrag konnte von −56.022,82 € auf −33.426,57 € reduziert werden (Tab. 6 und 7).

**Table 6:** 

Fallnr	Erlös (€)	Erlös neu (€)	Kosten (€)	Kosten neu (€)	DB (€)	DB neu (€)
1	47.539,76	43.016,06	40.440,18	39.082,18	7099,58	3933,88
2	17.594,41	13.716,91	23.541,59	19.993,19	−5947,18	−6276,28
3	11.116,78	11.116,78	20.246,01	18.701,19	−9129,23	−7584,41
4	11.308,23	11.308,23	13.932,11	12.886,23	−2623,88	−1578,00
5	14.754,72	12.967,90	23.074,57	18.485,17	−8319,85	−5517,27
6	5721,10	4422,16	12.411,70	8490,14	−6690,60	−4067,98
7	7345,24	7345,24	12.662,25	8373,30	−5317,01	−1028,06
8	27.495,16	23.566,04	45.209,26	41.203,98	−17.714,10	−17.637,94
9	11.898,53	11.898,53	10.335,46	6717,95	1563,07	5180,58
10	7345,24	7345,24	16.010,48	12.504,74	−8665,24	−5159,50
11	11.116,78	11.116,78	10.920,91	7532,35	195,87	3584,43
12	23.024,62	20.323,35	23.498,87	17.599,37	−474,25	2723,98
*Summe*	*196.260,57*	*178.143,22*	*252.283,39*	*211.569,79*	*−56.022,82*	*−33.426,57*

Die Begriffe Erlös/Kosten beziehen sich auf die Krankenhausperspektive.

*DB* Differenzbetrag

**Table 7:** 

Fallnr	Kosten (€)	Diff. [VD–OGVD] in Tagen	Tagessatz Normalstation (€)	VD ab Entlassfähigkeit in Tagen	Reduktion Kosten (€)
01	40.440,18	19	271,60	5	1358,00
02	23.541,59	27	236,56	15	3548,40
03	20.246,01	−4	257,47	6	1544,82
04	13.932,11	−2	261,47	4	1045,88
05	23.074,57	7	305,96	15	4589,40
06	12.411,70	6	230,68	17	3921,56
07	12.662,25	−9	285,93	15	4288,95
08	45.209,26	34	250,33	16	4005,28
09	10.335,46	−14	278,27	13	3617,51
10	16.010,48	−4	250,41	14	3505,74
11	10.920,91	−17	242,04	14	3388,56
12	23.498,87	11	235,98	25	5899,50
*Summe*	*252.283,39*	*–*	*–*	*–*	*40.713,60*

*Diff.* Differenz, *Fallnr* Fallnummer, *VD* Verweildauer, *OGVD* obere Grenzverweildauer

### Simulierte Kosten für „outpatient parenteral antibiotic therapy“

#### Kostenträger

Die Simulation führte in 11 Fällen zu Vorstellungen in 1 Quartal und in 1 Fall in 2 Quartalen. Die simulierten Gesamtkosten für die Kostenträger betrugen 1196 €. Die zusätzlichen Medikamentenkosten betrugen 56.199,28 € (Tab. 8).

**Table 8:** 

Fallnr	Kosten Poliklinik (€)	Kosten Antibiotika (€)	Gesamtkosten (€)
01	92	297,18	1485,90
02	92	265,38	3980,70
03	92	313,55	1881,30
04	92	313,55	1254,20
05	92	441,43	6621,45
06	92	441,43	7504,31
07	184	568,41	8526,15
08	92	265,38	4246,08
09	92	441,43	5738,59
10	92	377,12	5279,68
11	92	303,03	4242,42
12	92	265,38	6634,50
*Summe*	*1196*	*–*	*57.395,28*

*Fallnr* Fallnummer

#### Leistungserbringer

Die Gegenüberstellung der Kosten und Erlöse der Poliklinik resultiert in einem Defizit von 1579 € (Tab. 9).

**Table 9:** 

Fallnr	Erlös [E] Poliklinik (€)	Kosten [K] Poliklinik (€)	Diff. [E–K] Poliklinik (€)
01	92	75	17
02	92	450	−358
03	92	75	17
04	92	75	17
05	92	225	−133
06	92	225	−133
07	184	75	109
08	92	450	−358
09	92	150	−58
10	92	150	−58
11	92	150	−58
12	92	675	−583
*Summe*	*1196*	*2775*	*−1579*

*Diff.* Differenz, *Fallnr* Fallnummer

Die hier genannten Erlöse in der Poliklinik entsprechen sowohl den Erlösen aus Leistungsträgersicht als auch gleichermaßen den Kosten aus Kostenträgersicht. Die Kosten der Poliklinik – aus Leistungserbringerperspektive – entsprechen den kalkulierten Gesamtkosten einer Behandlung in der Poliklinik (ohne Zusatzleistungen wie Labor oder Röntgen)

### Gesamtkosten der „outpatient parenteral antibiotic therapy“

Aus Perspektive der Kostenträger traten zusätzlichen Kosten von 39.277,93 € auf.

Aus Perspektive der Leistungserbringer wurden 21.017,25 € mehr erwirtschaftet und 159 Bettentage gespart (Tab. 6, 7, 8 und 9).

## Diskussion

Eine sektorenübergreifende Behandlung von Patienten mit periprothetischen Gelenkinfektionen ist wünschenswert. Eine Möglichkeit stellt die Verschiebung erforderlicher parenteraler Antibiotikabehandlungen in den ambulanten Sektor dar. Neben psychosozialen Effekten könnte diese OPAT gegenüber der IPAT Kostenvorteile bieten. Eine umfassende Literaturrecherche zum Thema wurde kürzlich veröffentlicht [2]. Die Datenlage hinsichtlich der Kosten von IPAT und OPAT bei PJI ist jedoch gering. Alle Studien bis auf eine zeigten eine deutliche Kostenreduktion durch eine OPAT im Vergleich zur IPAT. Die Kosten der IPAT waren im Vergleich zur OPAT zwischen 1,1- und 17,3-fach höher. Daten aus Deutschland lagen bisher nicht vor. Die vorliegende Studie ist unserer Kenntnis nach die erste Kostenanalyse zur OPAT bei PJI in Deutschland.

Die Analyse der klinischen Daten in der vorliegenden Arbeit konnte in einem 1‑ Jahres-Zeitraum (2015) 12 Fälle identifizieren, die aus retrospektiver Betrachtung einer OPAT hätten zugeführt werden können. Der Anteil an den 131 Fällen mit der Diagnose T84.x betrug somit 9 %. Die vermeidbaren stationären Behandlungstage wurden konservativ auf 159 Tage geschätzt.

### Kosten der „inpatient parenteral antibiotic therapy“

Die Gesamtkosten der Behandlung im Krankenhaus lagen bei 252.283,39 € und standen Erlösen von 196.260,57 € gegenüber. Eine Reduktion der Behandlungsdauer hätte konsequenterweise zu einer geringeren Vergütung aufgrund kürzerer Verweildauern geführt. Die Reduktion der tatsächlichen Kosten belief sich dabei auf insgesamt 40.713,60 €. Die kalkulierten Gesamtkosten der eingesparten Behandlungstage aus Kostenträgerperspektive lagen bei insgesamt 18.117,35 €. Die Kosten pro Tag lagen bei durchschnittlich 299,96 € (Median 250,42 €; Spanne 216,49 –904,74 €). Im Kontext der Literatur zu den stationären Behandlungskosten der IPAT (Mittelwert 485,32 €; Median 439,76 €; Spanne 180,00–1125,00 €) lagen die Kosten in einem vergleichbaren Rahmen bzw. etwas darunter. Der negative Deckungsbetrag konnte somit in der Simulation von −56.022,82 € auf −33.426,57 € verringert werden, weitere Optimierungen wären jedoch notwendig, um in den positiven Bereich zu gelangen. Eine weitere Verkürzung der stationären Behandlung könnte hierzu z. B. durch eine progressivere Entlassungspolitik simuliert und untersucht werden.

Insgesamt ist erkennbar, dass die Besonderheiten der Abrechnung im stationären Sektor den Leistungserbringern Anreize zur Verkürzung der Liegezeiten geben.

### Kosten der „outpatient parenteral antibiotic therapy“

Der solitären Betrachtung der Kostenreduktion im stationären Sektor stehen die Kosten der ambulanten Behandlung gegenüber. Hierzu wurde in dieser Arbeit die Anbindung der Patienten an eine Heimtherapie mit Elastomerpumpen in Kombination mit Kontrollen in einer universitären Poliklinik als Kalkulationsgrundlage der OPAT-Kosten verwendet. Die Kosten der Antibiotika beliefen sich dabei auf durchschnittlich 4782,94 € pro Patient (1254,20–7504,31 €). Die Kosten pro Tag für Antibiotika lagen bei durchschnittlich 357,77 € (265,38–568,41 €). In Kombination mit den Pauschalbeträgen der Poliklinik lagen die Gesamtkosten der OPAT-Behandlung aus Perspektive der Kostenträger bei 57.395,28 €. Im Kontext der Literatur zu den ambulanten Behandlungskosten konnten die Therapiekosten pro Fall im internationalen Vergleich eingeordnet werden [2]. Die Tageskosten der OPAT in Deutschland hingegen liegen deutlich über den international publizierten Ergebnissen. Dies ist insbesondere den hohen Preisen der Antibiotika geschuldet.

### Gesamtkosten der „outpatient parenteral antibiotic therapy“

Erst durch die kombinierte Betrachtung der Kosten der ambulanten und stationären Behandlung kann eine Aussage zur möglichen Kostenreduktion der OPAT im Vergleich zur IPAT gelingen.

Aus Perspektive der Kostenträger entstünden durch eine OPAT relevante Kostenreduktionen des stationären Verlaufes. Demgegenüber entstanden zusätzliche Kosten durch die OPAT. Den Großteil der Kosten machten hierbei die Medikamentenkosten aus (98 %). Der Differenzbetrag von Kostenreduktion der IPAT und Kosten der OPAT belief sich auf 39.277,93 € zusätzlicher Kosten für die Kostenträger.

Aus Perspektive der Leistungserbringer standen einer Reduktion der Verluste um 33.426,57 € zusätzliche Kosten durch die OPAT in Höhe von 1579 € gegenüber. Es wurden somit für die Leistungserbringer direkte finanzielle Vorteile von 31.847,57 € durch eine simulierte OPAT generiert. Zusätzliche indirekte Vorteile z. B. durch die Neubelegung freier Betten (159 Bettentage) wurden hierbei noch nicht berücksichtigt.

Die unmittelbare Gegenüberstellung der IPAT und OPAT zeigte für das vorliegende Kollektiv, dass für Kostenträger aufgrund der hohen Medikamentenkosten 39.277,93 € Mehrkosten zu verzeichnen wären, derweil die Leistungserbringer einen Vorteil von 31.847,57 € erwirtschaften könnten.

Vor allem die Pauschalierung der Krankenhauskosten spielt hier eine Rolle für diese deutlichen Unterschiede. Werden im Krankenhaus die Kosten in der DRG mit eingeschlossen und sind die Pauschalen für eine Überschreitung der OGVD übersichtlich, so fallen doch zunächst durch eine OPAT höhere Kosten an. Zum einen werden die zuvor durch die DRG-Pauschale vergüteten Material‑, Medikamenten‑, Labor- und Personalkosten in den ambulanten Sektor verschoben und somit nicht mehr pauschal gedeckt, zum anderen ist eine Reduktion der stationären Kosten für den Kostenträger nur dann zu erwarten, wenn die Verweildauer (VD) über der OGVD läge. Läge die VD unter der OGVD, so entstünden für den Kostenträger grundsätzlich Mehrkosten durch eine OPAT. Aus Perspektive der Kostenträger ist daher die OPAT im Rahmen der aktuellen Kostenerstattung nur bedingt attraktiv.

Aus Perspektive der Leistungserbringer (Krankenhaus) stellt sich die Situation umgekehrt dar. Die pauschale Vergütung durch DRG resultiert in einem höheren Erlös pro Tag, wenn die Patienten früher als die OGVD entlassen werden. Zu Abschlägen käme es nur beim Unterschreiten der UGVD, was in diesen Fällen unwahrscheinlich wäre. Für die Krankenhäuser spielen zusätzlich die Opportunitätskosten eine relevante Rolle, da frei gewordene Betten neu belegt werden können und erneut Umsätze erwirtschaftet werden könnten. Eine Kalkulation dieser indirekten Kosten/Umsätze fand in dieser Arbeit nicht statt.

Weitere Aspekte, die für die OPAT berücksichtigt werden müssen, sind die möglichen Belastungen für die niedergelassenen Ärzte. Zum einen würden die mitunter stark ausgelasteten niedergelassenen Ärzte mit einem weiteren Aufgabenfeld betraut, zum anderen treten neben dem vermehrten Zeitaufwand weitere Belastungen auf. Die Verschiebung der gesamten Behandlung in den ambulanten Sektor erfordert ggf. auch die Rezeptierung von zusätzlichen Leistungen, die zuvor im stationären Sektor geleistet wurden. Dies könnten z. B. Physiotherapie, Ergotherapie, Lymphdrainage etc. sein. Die unmittelbaren Belastungen der niedergelassenen Ärzte schließen ärztliche Kontrolluntersuchungen, Laboruntersuchungen (Personal‑, Material- und Laborkosten) sowie die Rezeptierung der antibiotischen Therapie mit ein. Diese Belastungen könnten insbesondere bei zudem noch teuren Medikamenten eine immense Belastung für das Budget des niedergelassenen Arztes darstellen. Es ist daher notwendig, die niedergelassenen Ärzte eng in die Behandlung einzubinden und von den Vorteilen für die Patienten (und Gesellschaft) zu überzeugen. Alternativ könnten Patienten an spezialisierte Einrichtungen (integrierte OPAT-Ambulanz) angebunden werden. Diese müssten jedoch zunächst etabliert werden. Allerdings konnten Minton et al. nachweisen [8], dass diese Ambulanzen nicht nur von Vorteil sind, sondern durchaus auch mit gewissen Nachteilen einhergehen.

## Fazit für die Praxis

- Anhand der beispielhaften Kostenkalkulation einer universitären Poliklinik konnte gezeigt werden, dass die OPAT („outpatient parenteral antibiotic therapy“) für Leistungserbringer insgesamt finanziell vorteilhaft erscheint.
- Für den Kostenträger ist die OPAT allerdings potenziell mit einem finanziellen Mehraufwand verbunden. Dies resultiert insbesondere aus den ambulanten Medikamentenkosten.
- Für die Krankenhäuser als Leistungserbringer sind zudem die Opportunitätskosten interessant, da eine Neubelegung frei gewordener Betten ermöglicht wird und folglich erneut Umsätze erwirtschaftet werden könnten.
- Aufgrund der anzunehmenden Mehrbelastung erscheint die frühzeitige enge Einbindung der niedergelassenen Ärzte in die Planung einer OPAT zwingend erforderlich. Der niedergelassene Sektor sollte von den Vorteilen für die Patienten (und Gesellschaft) überzeugt werden.
- Eine Einführung von OPAT-Ambulanzen oder anderen Systemen, wie sie z. B. in Großbritannien etabliert sind, scheint eine sinnvolle Investition für die Zukunft zu sein.

## Abkürzungen

BBFW: Bundesbasisfallwert
BR: Bewertungsrelation
CIVAS: Community IntraVenous Antibiotic Study
DB: Differenzbetrag
DRG: Diagnosis Related Groups
GKV: gesetzliche Krankenversicherung
ICD-10 GM: Internationale statistische Klassifikation der Krankheiten und verwandter Gesundheitsprobleme, 10. Revision, German Modification
InEK: Instituts für das Entgeltsystem im Krankenhaus
IPAT: inpatient parenteral antibiotic therapy
IQTiG: Institut für Qualitätssicherung und Transparenz im Gesundheitswesen
KTEP: Knietotalendoprothese
MVD: Mindestverweildauer
OGVD: obere Grenzverweildauer
OPAT: outpatient parenteral antibiotic therapy
PICC: peripherally inserted central venous catheter
PJI: periprosthetic joint infections
UGVD: untere Grenzverweildauer
VD: Verweildauer
ZE: Zusatzentgelte
ZVK: zentraler Venenkatheter
